# Glucocorticoid-resistant polymyalgia rheumatica: pretreatment characteristics and tocilizumab therapy

**DOI:** 10.1007/s10067-014-2650-y

**Published:** 2014-05-08

**Authors:** Shunsuke Mori, Yukinori Koga

**Affiliations:** 1 Department of Rheumatology, Clinical Research Center for Rheumatic Disease, NHO Kumamoto Saishunsou National Hospital, 2659 Suya, Kohshi, Kumamoto, 861-1196 Japan; 2 Department of Radiology, Clinical Research Center for Rheumatic Disease, NHO Kumamoto Saishunsou National Hospital, Kumamoto, Japan

**Keywords:** Glucocorticoid resistance, Interleukin-6, PMR activity score, Polymyalgia rheumatica, Tocilizumab

## Abstract

Treatment with glucocorticoid (GC) is the preferred therapy for polymyalgia rheumatica (PMR), but some patients show poor responses to the initial GC regimen or experience flares on GC tapering. Alternative therapies for patients with GC resistance have not yet been established. To evaluate pretreatment characteristics and therapeutic outcomes of GC-resistant PMR, we followed all patients who had been diagnosed with PMR between October 2007 and February 2013, according to our standardized protocol. GC-resistant patients were defined as those who had responded poorly to the initial GC regimen (15 mg/day of prednisolone) or those who had responded to the initial regimen but had experienced a flare upon GC tapering to 5 mg/day of the maintenance dose or within the first 6 months of maintenance therapy. Out of 23 patients, nine were found to be GC-resistant cases and the others were GC responders. Baseline values of PMR activity score and its components, especially the ability to elevate the upper limbs (EUL), were significantly higher in GC-resistant patients compared with GC responders. The additional use of methotrexate (MTX, five cases), salazosulfapyridine (one case), and tocilizumab (TCZ, three cases) was effective for GC-resistant patients, although 13 to 39 weeks were required for the achievement of remission. We report the three GC-resistant cases in which TCZ was added to GC therapy with or without MTX. We also review the medical literature on the use of TCZ as of January 31, 2014 and discuss the utility of TCZ in the treatment of GC-resistant PMR.

## Introduction

Polymyalgia rheumatica (PMR) is an inflammatory condition of unknown etiology that is characterized by pain and morning stiffness in the neck, shoulder, and pelvic girdles. It affects people over 50 years of age. Constitutional symptoms such as low-grade fever, fatigue, and weight loss, as well as increased levels of acute inflammatory reactants, are present in most patients [1]. Although the proximal symptoms are prominent, distal musculoskeletal manifestations are also frequently observed in PMR patients [2]. Articular and periarticular synovitis appears to be commonly and primarily responsible for the proximal and distal musculoskeletal symptoms of PMR [3].

Usually, PMR is dramatically improved with a once-daily low dose of glucocorticoids (GCs). Clinical symptoms resolve completely or nearly completely within one to a few days in response to 15- to 20-mg daily prednisolone or equivalent, and acute inflammatory reactants are normalized within 4 weeks [4]. However, approximately half of PMR patients experience a flare of disease activity upon GC tapering or discontinuation [5–7]. Patients with a chronic, repeated relapsing course may require GC therapy for several years, which can cause undesirable GC-related adverse events such as osteoporotic fracture, diabetes mellitus, hypertension, infections, gastrointestinal bleeding, and other conditions. A population-based study showed that approximately 65 % of PMR patients had at least one GC-related serious adverse event [8]. In addition, some patients have only a partial response or do not respond to the initial GC regimen. Hutchings et al. reported that 55 % of patients showed poor or partial response to 3-week GC therapy [9]. Currently, it is difficult to predict which patients are most at risk for developing such GC resistance.

Furthermore, as there are no guidelines for the management of GC-resistant PMR patients, alternative therapies may be considered. Immunosuppressive agents such as methotrexate (MTX) and biological agents such as anti-tumor necrosis factor-α (anti-TNFα) agents and anti-interleukin-6 (anti-IL-6) receptor antibody tocilizumab (TCZ) may be possible candidates as adjunctive therapies.

In this study, we followed all patients who had been diagnosed with PMR at our hospital from October 2007 to February 2013, according to our standardized protocol for PMR management, and evaluated pretreatment clinical features and therapeutic outcomes of GC-resistant cases. Among these cases, we report three patients who achieved remission by the addition of TCZ. We also review the medical literature on TCZ use for PMR that was isolated through a search of the PubMed database as of January 31, 2014.

## Patients and methods

### Patients

We followed all patients who had been diagnosed with PMR at our hospital from October 2007 to February 2013 according to our PMR management protocol. Diagnosis had been made on the basis of Bird’s criteria for PMR [10]. We confirmed that all patients fulfilled the 2012 provisional classification criteria for PMR by the European League Against Rheumatism (EULAR) and the American College of Rheumatology (ACR) [1]. There were no clinical symptoms or signs to raise clinical suspicion of giant cell arthritis (GCA), other systemic vasculitis, rheumatoid arthritis, or seronegative spondylarthropathy. There was no muscle weakness or atrophy. Peripheral joint involvement was seen in four patients. Rheumatoid factor (RF), anti-citrullinated peptide antibodies (anti-CCP Abs), antinuclear antibodies (ANAs), and anti-neutrophil cytoplasmic antibodies (ANCAs) were all negative. No increases in muscular enzymes were detected. No patients had any clinical evidence suggesting active cancer or infectious disease. Inflammatory markers were determined at presentation and every 2 to 4 weeks during the follow-up period. All patients were followed for at least 1 year.

### Protocol of PMR management

All PMR patients were treated at our hospital according to the following protocol for PMR management. Patients started with an initial regimen of 15 mg of prednisolone daily as a single morning dose. To evaluate pretreatment factors associated with the development of GC resistance, the fixed initial dose was used for all patients. Based on data from the only randomized controlled studies on initial prednisolone doses, we determined 15 mg/day of prednisolone as the initial regimen [11, 12]. After 2 to 3 weeks, the daily dose was reduced by 2.5 mg every 2 weeks to 10 mg/day as long as symptoms remained improved. The daily dose was then tapered by 1 to 2 mg every month until a maintenance dosage of 5 mg/day was reached if remission persisted. The maintenance therapy was continued for at least 6 months. Further reduction and suspension of GC were left to the discretion of the treating rheumatologist. If the patient did not respond to the initial GC regimen and no alternative diagnosis was made, the addition of other immunosuppressive agents was considered. If the patient experienced a flare on tapering to the maintenance dose of 5 mg/day, the addition of other immunosuppressive agents was considered. If the patient experienced a flare when reducing the prednisolone dose below 5 mg/day, an increase to the previously effective dosage was made.

### Definition of GC resistance

GC responders were defined as patients who had responded well to the initial GC dose, tolerated a reduction to the maintenance dose of 5 mg/day, and thereafter maintained remission for at least 6 months. GC-resistant patients were defined as those who had responded poorly to the initial GC regimen or those who had responded to the initial regimen but had experienced a flare upon GC tapering to the maintenance dose of 5 mg/day or within the first 6 months of the maintenance therapy. A flare was defined as an exacerbation or reappearance of PMR symptoms (aching and stiffness at the shoulder girdle, pelvic girdle, or both) associated with abnormal C-reactive protein (CRP, >0.5 mg/dl) level, erythrocyte sedimentation rate (ESR, >30 mm/h), or both. Remission was defined as achieving a PMR activity score (PMR-AS) of 0 to 1.5 [13].

### Calculation of PMR-AS

The PMR-AS was calculated according to the equation CRP (mg/dl) + the visual analog scale for pain (VAS-p, 0–10) + the visual analog scale for physician’s assessment (VAS-ph, 0–10) + morning stiffness (MST, min) × 0.1 + ability to elevate the upper limbs (EUL, 0–3) [14].

## Clinical characteristics of GC-resistant PMR patients

Fourteen patients were GC responders and nine were GC-resistant. No GC-related severe adverse events were seen during the follow-up period. All GC responders achieved remission within 2 to 6 weeks and maintained it for more than 6 months. Among these patients, seven maintained remission for more than 6 months without prednisolone. In GC-resistant patients, three did not respond to the initial GC regimen and six did not tolerate GC tapering. The addition of MTX and salazosulfapyridine (SASP) was effective for five and one patients, respectively, though 13 to 32 weeks were required for the achievement of remission. Among these six patients, three successfully discontinued all agents for PMR therapy and maintained remission for more than 6 months. The other three maintained remission for more than 6 months with 5 mg/day of prednisolone. The remaining three patients received TCZ with or without MTX (see next section). Clinical characteristics of GC responders and GC-resistant patients at baseline are shown in Table 1. Pretreatment values of the PMR-AS and its components, especially EUL, were significantly higher in GC-resistant patients compared with GC responders. In addition, GC-resistant patients were more likely to take a longer time to achieve first remission.

**Table 1:** Baseline characteristics of PMR patients: comparison of GC responders and GC-resistant patients

	GC responder group (*n* = 14)	GC-resistant group (*n* = 9)	*p* values^a^
Age, median (IQR) (years)	65 (61.3–77.8)	73 (67–77)	0.49
Male/female	8/6	2/7	0.11
Duration of symptoms before therapy, median (IQR) (weeks)	2 (2–2)	2 (2–4)	0.33
C-reactive protein level, median (IQR) (mg/dl)	4.8 (4.0–9.9)	11.5 (8.0–13.6)	0.018
Erythrocyte sedimentation rate, median (IQR) (mm/h)	78.5 (51.3–82)	100 (81–103)	0.068
PMR activity score, median (IQR)	23.8 (22.2–35.5)	48 (44.5–66)	0.0098
Patient’s pain assessment (VAS 0–10), median (IQR)	6.5 (4.3–7.8)	9 (7–10)	0.014
Physician’s global assessment (VAS 0–10), median (IQR)	6 (4.3–7)	9 (7–10)	0.033
Morning stiffness (min), median (IQR)	60 (60–120)	240 (120–300)	0.019
EUL (0–3), median (IQR)	1 (1–2)	3 (2–3)	0.0026
Peripheral arthritis^b^, numbers of patients (%)	0	4 (44.4)	0.014
Body weight (kg), median (mean ± SD)	55.4 ± 9.6	53.1 ± 10.3	0.30
Duration to first remission (weeks), median (IQR)	2 (2–2)	30 (26–34)	<0.0005

*PMR* polymyalgia rheumatica, *GC* glucocorticoid, *IQR* interquartile range, *VAS* visual analog score, *EUL* ability to elevate the upper limbs

^a^Clinical characteristics of GC responders and GC-resistant patients were compared using the Mann-Whitney *U* test for continuous variables and *χ* ^2^ test and Fisher’s exact probability test for categorical variables

^b^Peripheral arthritis includes knees, wrists, ankles, elbows, hands, and feet

## Case reports of GC-resistant and TCZ-treated PMR

We present three GC-resistant PMR patients who received TCZ therapy. Cases 1 and 2 were MTX-resistant and SASP-resistant PMR, respectively. In case 3, PMR signs and symptoms were markedly aggravated during the initial GC therapy.

### Case 1

A 55-year-old woman visited our hospital with chief complaints of recent onset (within 2 weeks) of bilateral shoulder and neck pain, bilateral hip pain, and morning stiffness lasting 4 h. Arms and thighs were tender to touch. Shoulder active motion was grossly limited (EUL, grade 3). VAS-p and VAS-ph were 7, and CRP and ESR levels were 6.95 mg/dl and 97 mm/h, respectively. The white blood cell (WBC) count was 7,400/ml, and the PMR-AS was 48. Fat suppression magnetic resonance imaging (MRI) of the shoulder showed evidence of subacromial and subdeltoid bursitis, glenohumeral joint synovitis, and biceps tenosynovitis.

The patient started with 15 mg/day of oral prednisolone. At week 3 of treatment, the patient’s PMR-AS was decreased to 9 and GC tapering was started by 2.5 mg every 2 weeks. At week 5, the PMR-AS remained unchanged (9.7). Six milligrams per week of MTX was added to treatment with 10 mg/day of prednisolone. Since the PMR-AS continued to worsen (33.1 at week 13), the MTX dose was increased to 8 mg/week. At week 17, PMR-AS was not improved (35.1) and CRP was 6.73 mg/dl. TCZ therapy (8 mg/kg, every 4 weeks, intravenously) was introduced to the patient in combination with MTX (8 mg/week) and prednisolone (7.5 mg/day). One month later, the patient achieved remission (PMR-AS, 0.1). Further three infusions of TCZ were given. At week 29, prednisolone and MTX was reduced to 5 mg/day and 6 mg/week, respectively. The patient maintained remission for more than 8 months with this regimen.

### Case 2

A 67-year-old woman visited our hospital because of recent onset (within 2 weeks) of bilateral shoulder and neck pain, and morning stiffness lasting 4 h. The first physical examination showed reduced range of motion of the shoulders and neck (EUL, grade 3). Arms and thighs were tender to touch; VAS-p and VAS-ph were 9; and CRP and ESR levels were 11.47 mg/dl and 103 mm/h, respectively. The WBC count was 8,040/ml, and the PMR-AS was 56.5. Fat suppression MRI of the shoulder showed evidence of subacromial bursitis, glenohumeral joint synovitis, and biceps tenosynovitis.

The patient started with 15 mg/day of oral prednisolone. At week 3 of treatment, the patient’s clinical symptoms improved with decreased CRP (1.5 mg/dl), and prednisolone tapering was started by 2.5 mg every 2 weeks until 10 mg/day. At week 5, the CRP level was 1.28, and it continued to decrease during the next month. At week 11, however, the CRP level rose to 1.82 mg/dl and therefore SASP (1 g/day) was added to treatment with 10 mg/day of prednisolone. At week 13, PMR symptoms remained aggravated and CRP was increased to 6.59. Prednisolone was increased to 15 mg/day. SASP was discontinued because of abnormal values of serum hepatic enzymes. At week 17, the patient achieved remission (CRP, 0.1 mg/dl; PMR-AS, 0.5) and maintained remission with 5 mg/day of prednisolone for 3 months. At week 29, she experienced a flare (PMR-AS, 11.3; CRP, 2.25 mg/dl). TCZ therapy (8 mg/kg, every 4 weeks, intravenously) was added to 5 mg/day of prednisolone. One month later, the patient achieved remission (PMR-AS, 0.1). An additional infusion of TCZ was given. Prednisolone was then tapered off over 6 months.

### Case 3

A 73-year-old woman consulted a neighboring clinic because of recent onset (within 2 weeks) of bilateral shoulder and neck pain. Since PMR was suspected, 10 mg/day of prednisolone was prescribed. Two days later, the patient was referred to our hospital for aggravation of symptoms. The patient complained of bilateral shoulder, hip, and neck pain and morning stiffness lasting 6 h. Active movements of the shoulders and neck were markedly restricted (EUL, grade 3). Arms and thighs were tender to touch; VAS-p and VAS-ph were both 10; and the values of CRP and ESR were 14.95 mg/dl and 81 mm/h, respectively. The WBC count was 16,200/ml, and the PMR-AS was 74. Fat suppression MRI of the shoulder showed evidence of glenohumeral joint synovitis and biceps tenosynovitis.

The patient started with 15 mg/day of oral prednisolone. Since the patient’s symptoms were aggravated for 2 weeks of this therapy (CRP, 14.99 mg/dl; PMR-AS, 97), TCZ therapy was added (8 mg/kg, every 4 weeks) to treatment with 15 mg/day of prednisolone. One month after the first TCZ infusion, the CRP was 8.92 mg/dl and PMR-AS was 59. Because of a suspected non-PMR diagnosis, we examined blood cultures, gallium scintigraphy, computer tomography of the abdomen and pelvis with contrast, and upper gastrointestinal endoscopy, but we found no evidence of active infections or malignancies. We also confirmed that there were no clinical symptoms or signs of GCA and then restarted TCZ therapy (162 mg, every 2 weeks, subcutaneously) with 10 mg/day of prednisolone. Prednisolone was tapered by 1 mg every month. After six injections of TCZ, disease activity was still within the median range (PMR-AS, 14) despite the normalization of CRP. MTX (6 mg/week) was added to treatment with TCZ and prednisolone (5 mg/day). Through 3 months of this treatment, the patient achieved remission (PMR-AS, 1.1).

## Literature review on TCZ treatment for PMR patients

By performing a search of the medical literature as of January 31, 2014 using the terms “polymyalgia rheumatica,” “tocilizumab,” and “anti-IL-6 receptor,” we identified 13 cases where PMR patients were treated with TCZ alone or in combination with GCs: five were pure PMR cases and eight were GCA-associated PMR cases [15–21]. One patient with pure PMR had unclassified aortitis without clinical manifestations related to large-vessel vasculitis such as GCA or Takayasu arteritis [19]. Clinical features and therapeutic outcomes of these cases, including the three pure PMR cases in the present study, are summarized in Table 2 (cases 1–8: pure PMR) and Table 3 (cases 9–16: GCA-associated PMR).

**Table 2:** Clinical characteristics and therapeutic outcomes of pure PMR cases in patients receiving TCZ therapy

Case no.	Age/sex	PMR features	TCZ therapy	Drugs at the start of TCZ therapy	Outcomes at the time of TCZ end	Time to achieve remission	Drugs at the end of TCZ therapy	Ref. no.
1	55/F	GC-resistant MTX-resistant	8 mg/kg/month (4 infusions)	MTX (8 mg/week) PSL (7.5 mg/day)	Remission (PMR-AS, 0.1)	1 month	MTX (6 mg/week) PSL (5 mg/day)	Present study
2	67/F	GC-resistant SASP-resistant	8 mg/kg/month (2 infusions)	PSL (5 mg/day)	Remission (PMR-AS, 0.1)	1 month	PSL (3 mg/day)	Present study
3	73/F	GC-resistant	8 mg/kg/month (1 infusion) 162 mg/2 weeks (12 injections)	PSL (15 mg/day)	Remission (PMR-AS, 1.1)	7 months	MTX (6 mg/week) PSL (5 mg/day)	Present study
4	87/F	New-onset GC-naive	8 mg/kg/month (2 infusions)	None	Poor response (PMR-AS, 10.4)	No remission	None^a^	[20]
5	62/M	GC-resistant	8 mg/kg/month (ND)	mPSL pulse (3 g in total) then TCZ start	Remission^b^	ND	PSL (10 mg/day)	[19]
6	65/F	GC-resistant	8 mg/kg/month (12 infusions)	PSL (10 mg/day)	Remission (PMR-AS, 0.74)	5 months	PSL (6 mg/day)	[15]
7	ND	GC-resistant	8 mg/kg/month (4 infusions)	None	Remission^b^	2–3 months	None	[18]
8	62/F	GC responder (adverse event) MTX-resistant ETN-resistant	8 mg/kg/month (12 infusions)	None	Remission (PMR-AS, 1.5)	3 months	None	[21]

*TCZ* tocilizumab, *MTX* methotrexate, *SASP* salazosulfapyridine, *PSL* prednisolone, *mPSL* methylprednisolone, *ETN* etanercept, *PMR* polymyalgia rheumatica, *ND* not described

^a^After discontinuation of TCZ, the patient was started on treatment with 15 mg/day of PSL. The disease activity was subsequently well-controlled with few flares and she remained in complete remission without GC

^b^Remission was diagnosed based on laboratory signs and symptoms

**Table 3:** Clinical characteristics and therapeutic outcomes of GCA-associated PMR cases in patients during TCZ therapy

Case no.	Age/sex	PMR features	GCA associations	TCZ therapy	Drugs at the start of TCZ therapy	Outcomes at the end of TCZ therapy	Time to remission	Drugs at the end of TCZ therapy	Ref. no.
9	79/F	New-onset GC-naive	5 years ago Remission	8 mg/kg/month (6 infusions)	None	Partial response^a^ (PMR-AS, 6.9)	No remission	None	[20]
10	63/F	GC-resistant MTX-resistant LEF-resistant AZA-resistant	Active	8 mg/kg/month (4 infusions) 4 mg/kg/month (2 infusion)	PSL (20 mg/day)	Remission (VAS-p and CRP)	1 month	PSL (3.5 mg/day)	[16]
11	ND	GC-resistant CYC-resistant	Active	8 mg/kg/month (11 infusions)	PSL (30 mg/day)	Remission^c^	2–3 months	None	[18]
12	ND	GC-resistant MTX-resistant ADM-resistant ETN-resistant	Previous GCA	4 mg/kg/month (7 infusions)	PSL (15 mg/day)	Remission^c^	2–3 months	PSL (6 mg/day)	[18]
13	63/M	New-onset GC-naive	Active	8 mg/kg/month^b^ (ND)	None	Remission (VAS, CRP, ESR)	Within 2 months	None	[17]
14	73/M	New-onset GC-naive	Active	8 mg/kg/month^b^ (ND)	None	Remission (VAS, CRP, ESR)	Within 2 months	None	[17]
15	79/F	GC-resistant MTX-resistant	Active	8 mg/kg/month^b^ (ND)	PSL (10 mg/day)	Remission (VAS, CRP, ESR)	Within 2 months	None	[17]
16	64/F	GC-resistant	Active	8 mg/kg/month^b^ (ND)	PSL (27.5 mg/day)	Remission (VAS, CRP, ESR)	Within 2 months	PSL (5 mg/day)	[17]

*TCZ* tocilizumab, *MTX* methotrexate, *SASP* salazosulfapyridine, *PSL* prednisolone, *mPSL* methylprednisolone, *ETN* etanercept, *PMR* polymyalgia rheumatica, *CYC* cyclophosphamide, *ADM* Adriamycin, *AZA* azacitidine, *LEF* leflunomide, *ND* not described

^a^Five months after the last infusion of TCZ, the patient experienced a relapse, but she achieved remission with 8 mg/day of mPSL. A complete remission was maintained by treatment with 2 mg/day of mPSL

^b^For the first month, TCZ was given every other week

^c^Remission was diagnosed based on laboratory signs and symptoms

In pure PMR cases, all patients except case 3 received 8 mg/kg of TCZ monthly. In case 3, the patient received a subcutaneous TCZ injection (162 mg) every 2 weeks. One patient (case 4) was newly diagnosed and had never received GCs before starting TCZ therapy. Another patient responded well to GC therapy but suffered from GC-related adverse events (case 8). This patient showed MTX and etanercept resistance. The remaining six patients were GC-resistant cases. Cases 1 and 2 showed MTX and SASP resistance, respectively. In cases 3 and 4, the patients had poor responses to TCZ therapy (case 3, 4 months of combination therapy with prednisolone; case 4, 2 months of monotherapy). In case 3, however, the addition of MTX resulted in remission. In case 4, after the discontinuation of TCZ, the patient achieved remission by prednisolone alone. In case 1, the addition of TCZ to treatment with MTX and prednisolone produced remission within 1 month. In cases 2, 5, and 6, the addition of TCZ to GC therapy markedly improved clinical symptoms and laboratory data. The GC dose was successfully reduced during TCZ therapy (cases 1–3 and 5–6). Two patients achieved remission with TCZ monotherapy (cases 7 and 8).

In eight patients with GCA-associated PMR, seven were treated with 8 mg/kg of TCZ monthly and one received 4 mg/kg monthly (case 12). Three cases were GC naive (cases 9, 13, and 14). Five patients achieved remission within 3 months of TCZ therapy combined with prednisolone and tolerated the GC tapering during the TCZ use (cases 10–12, 15, and 16). Among these, two patients successfully discontinued prednisolone (cases 11 and 15). Two patients achieved remission with TCZ monotherapy (cases 13 and 14). The remaining one had partial responses to six infusions of TCZ without GC (case 9).

These data suggest the utility of TCZ as a GC-sparing agent or as monotherapy for PMR patients. The introduction of TCZ to GC-resistant PMR patients may reduce cumulative GC doses or even replace GC therapy, which may result in a decreased risk of GC-related adverse events. It is unclear whether TCZ may be the first-line agent for the treatment of PMR. Given the high cost of TCZ therapy, GC therapy continues to be the first choice for PMR patients.

## Discussion

IL-6 is produced at the site of inflammation and plays a key role in the acute phase response. The correlation of serum levels of IL-6 and PMR activity during GC therapy has been examined over the past two decades. The majority of studies have indicated that IL-6 levels are significantly higher in active PMR patients compared with those in healthy controls [22–29] and that the administration of GCs significantly reduces IL-6 levels [22–25, 27–29]. Serum levels of IL-6 in PMR patients were significantly increased at the time of clinical relapse compared with those in patients in remission [30]. Several studies showed that persistently elevated levels of serum IL-6 during GC therapy are significantly associated with an increased risk of relapse and recurrence of PMR activity as well as a prolonged course with a need for increased prednisolone dose [5, 31]. A high level of serum soluble IL-6 receptor was identified as a potential prognostic marker for relapse in PMR patients [7]. These data support the idea that IL-6 is an important mediator in the pathogenesis of PMR. Through a search of the Medline database between 1980 and April 2008, Martinez-Taboada et al. indicated that PMR is characterized by a hyperproduction of IL-6 and that GC therapy is followed by a significant decrease in serum IL-6 [32].

In the present study, the five patients with GC-resistant PMR responded well to the addition of 6 to 8 mg/week of MTX to GC therapy and maintained remission for more than 6 months with or without 5 mg/day prednisolone, although one patient failed to achieve remission with the addition of MTX (case 1 in Table 2). To examine the GC-sparing effect of MTX, two randomized, double-blinded, placebo-controlled trials were performed. One study reported that the concomitant use of MTX in newly diagnosed PMR patients was associated with shorter GC treatment and GC sparing, when given for at least 1 year at a dose of at least 10 mg/day [33]. A long-term follow-up of these patients indicated that MTX does not decrease the incidence of GC-related adverse events, however [34]. Another study indicated that there were no differences between the MTX and placebo groups in active, untreated PMR patients in terms of time to achieve remission, duration of remission, number of flares, cumulative GC doses, or GC-related adverse events [35]. However, these studies may have included a considerable number of GC responders (patients who respond well to GC therapy and tolerate GC reduction without the use of MTX), which may lead to an underestimation of the GC-sparing effect of MTX. The GC-sparing effect of MTX should be further examined in randomized, controlled studies for GC-resistant PMR patients.

Several pretreatment parameters have been suggested as the risk factors for the development of GC resistance. Relapses may occur more frequently in women [36, 37], and female sex and increased age were reported to be risk factors for requiring longer GC therapy [38]. In addition, increased levels of inflammatory parameters such as CRP and ESR at baseline have been reported as risk factors for relapse/recurrence and prolonged GC therapy [31, 37–40]. However, some studies reported that there were no clinical or laboratory features significantly associated with relapses or prolonged GC therapy [41–43]. Using multivariate analysis, Cimmino et al. recently indicated that the only factor predicting a good response to GCs is low weight, when PMR patients are treated with an initial dose of 12.5 mg/day prednisolone (responders 67.4 kg vs. non-responders 78.5 kg) [44]. In our cohort, there was no difference in body weight between the two groups, which may be explained by the lower mean body weight of our patients (55.1 vs. 53.1 kg).

In the present study, the pretreatment values of PMR-AS and its five components, especially EUL, were significantly higher in GC-resistant patients. The PMR-AS, which was developed by Leeb and Bird based on a core set of five variables composing EULAR response criteria for PMR (CRP, VAS-p, VAS-ph, MST, and EUL), provides an easily applicable and valid tool for monitoring PMR activity in everyday practice. In combination with the EULAR response criteria, it provides a better description of treatment response [14, 45]. In a prospective cohort study, Binard et al. showed that a diagnosis of disease flare is strongly associated with PMR-AS values ≥9.35 and their changes between two visits to the physician ≥6.6 [46]. The changes of disease activity over time seem even more relevant and perform better than the absolute PMR-AS values when predicting a need of the GC dose increase [46, 47]. These studies showed the utility of this scoring system in monitoring PMR activity and making clinical and therapeutic decisions for individual patients. In addition to monitoring PMR activity, our findings have suggested that these parameters may be helpful in predicting the development of GC resistance in individual patients at the time of diagnosis and therefore identifying patients who will require additional therapy for the management of PMR.

## Conclusion

Although GCs are still the mainstay in PMR treatment, the use of alternative immunosuppressive agents should be considered for GC-resistant patients in order to minimize the cumulative GC exposure. Given the involvement of IL-6 in PMR pathogenesis, TCZ may be one of the most promising therapeutic options for this inflammatory disorder. The pretreatment values of PMR-AS and its components may be helpful in predicting which patient is most at risk for developing GC resistance. Regarding the prognostic utility of baseline PMR-AS values and the utility of TCZ therapy for GC-resistant PMR, we require careful scrutiny in large-scale follow-up studies where patients are treated according to standardized guidelines for PMR management.
